# Maternal inheritance of deltamethrin resistance in the salmon louse *Lepeophtheirus salmonis* (Krøyer) is associated with unique mtDNA haplotypes

**DOI:** 10.1371/journal.pone.0180625

**Published:** 2017-07-12

**Authors:** Greta Carmona-Antoñanzas, Michaël Bekaert, Joseph L. Humble, Sally Boyd, William Roy, David I. Bassett, Ross D. Houston, Karim Gharbi, James E. Bron, Armin Sturm

**Affiliations:** 1 Institute of Aquaculture, Faculty of Natural Sciences, University of Stirling, Stirling, Scotland, United Kingdom; 2 The Roslin Institute and Royal (Dick) School of Veterinary Studies, University of Edinburgh, Edinburgh, Scotland, United Kingdom; 3 School of Biological Sciences, University of Edinburgh, Edinburgh, Scotland, United Kingdom; University of the Sunshine Coast, AUSTRALIA

## Abstract

Parasitic infections by the salmon louse, *Lepeophtheirus salmonis* (Krøyer), cause huge economic damage in salmon farming in the northern hemisphere, with combined treatment costs and production losses in 2014 having been estimated at US$ 350 million for Norway (annual production 1.25 million tonnes). The control of *L*. *salmonis* relies significantly on medicinal treatments, supplemented by non-pharmacological approaches. However, efficacy losses have been reported for several delousing agents, including the pyrethroid deltamethrin. The aim of the present study was to analyse the genetic basis of deltamethrin resistance in *L*. *salmonis*. Deltamethrin median effective concentrations (EC_50_) were 0.28 μg L^-1^ in the drug susceptible *L*. *salmonis* strain IoA-00 and 40.1 μg L^-1^ in the pyrethroid resistant strain IoA-02. IoA-00 and IoA-02 were crossed to produce families spanning one parental and three filial generations (P0, F1-F3). In three families derived from P0 crosses between an IoA-00 sire and an IoA-02 dam, 98.8% of F2 parasites (n = 173) were resistant, *i*.*e*. remained unaffected after exposure to 2.0 μg L^-1^ deltamethrin. F3 parasites from these crosses showed a deltamethrin EC_50_ of 9.66 μg L^-1^. In two families of the inverse orientation at P0 (IoA-02 sire x IoA-00 dam), 16.7% of F2 parasites were resistant (n = 84), while the deltamethrin EC_50_ in F3 animals was 0.26 μg L^-1^. The results revealed a predominantly maternal inheritance of deltamethrin resistance. The 15,947-nt mitochondrial genome was sequenced and compared among six unrelated *L*. *salmonis* strains and parasites sampled from wild salmon in 2010. IoA-02 and three further deltamethrin resistant strains, established from isolates originating from different regions of Scotland, showed almost identical mitochondrial haplotypes. In contrast, the mitochondrial genome was variable among susceptible strains and *L*. *salmonis* from wild hosts. Deltamethrin caused toxicity and depletion of whole body ATP levels in IoA-00 but not IoA-02 parasites. The maternal inheritance of deltamethrin resistance and its association with mitochondrial haplotypes suggests that pyrethroid toxicity in *L*. *salmonis* may involve molecular targets encoded by mitochondrial genes.

## Introduction

Globally, insecticides play an important role in the control of arthropod disease vectors and crop-infesting pests. However, the development of insecticide resistance reduces the options available for chemical control, which potentially threatens global human health and food security [1–3]. Insecticide resistance in arthropods is based on the emergence and subsequent enrichment of resistance alleles in a population’s gene pool due to selective pressure caused by use of the same or similar acting control agent(s) over several generations [4]. The main molecular mechanisms of insecticide resistance include target site changes disrupting the control agent’s efficacy and the enhanced expression of detoxification mechanisms decreasing internal exposure to the insecticide [5,6].

Pyrethroids are synthetic insecticides that have been developed as improved structural analogues of the plant-derived pyrethrins [7–9]. Similarly to dichlorodiphenyltrichloroethane (DDT) and pyrethrins, the insecticidal activity of pyrethroids involves binding to and delaying the closing of arthropod neuronal voltage gated sodium channels (Na_v_) [10,11]. Despite some decline in usage since the 1990s, pyrethroids still achieved a 17% share of global insecticide sales in 2013, which makes them the second most common class of current insecticides [12,13]. Resistance to pyrethroids has developed in many arthropod species, and is frequently based on either or both of two main molecular mechanisms. Knock-down resistance (*kdr*) is based on specific non-synonymous point mutations of Na_v_ that render the channel insensitive to pyrethroids and further confer cross-resistance to DDT [11,14]. *Kdr* has been detected in pyrethroid-resistant isolates of numerous unrelated insect species [11,14,15], providing an example of convergent evolution. By contrast, metabolic resistance is based on the enhanced detoxification of pyrethroids, which in most cases is achieved through increased expression of one or several cytochrome P450s [2,16,17]. Metabolic pyrethroid resistance can further involve up-regulation of members of other multi-gene families with roles in biochemical defence, including carboxylesterases, glutathione S-transferases and ABC transporters [18–21].

Since the mid-1990s, the pyrethroids cypermethrin and deltamethrin have been employed as veterinary medicines prescribed to treat farmed Atlantic salmon (*Salmo salar* Linnaeus, 1758) suffering from parasitic infections by sea lice (Copepoda: Caligidae) [22,23]. Caligids feed on the skin tissues and blood of the host fish, causing lesions, osmoregulatory imbalance, growth and immune suppression, secondary infections and, at high parasite densities, potentially death [24]. Reproducing female sea lice carry eggs that hatch to free-swimming nauplial larvae, which require 2–9 days to develop to the infectious copepodid stage, depending on ambient temperatures [25]. Increasingly complex marine hydrographic models have been built to predict the dispersal of sea louse larvae, suggesting transmission can occur over distances of up to ~30 km [26,27]. In the Northern hemisphere, most problematic caligid infections of farmed salmon involve the salmon louse (*Lepeophtheirus salmonis*, (Krøyer, 1837)), with rarer problems caused by a smaller species, *Caligus elongatus* Nordmann, 1832. In Chile, by contrast, *Caligus rogercresseyi* Boxshall & Bravo, 2000 causes major damage in commercial salmon production [28,29]. The economic costs of sea lice control to the global salmon farming industry have been estimated at more than €300 million per annum in 2008 [28]. Treatment costs have since then increased by >60% in Norway [30], due to increase in production but also resistance formation, and can now reach 10% of the total production costs of farmed salmon [31]. Resistance of *L*. *salmonis* to pyrethroids, which was initially reported in the mid-2000s [32], is currently widespread in Norway [22]. While roles for cytochrome P450 and *kdr*-type mutations as determinants of pyrethroid resistance in *L*. *salmonis* have been investigated [33,34], results of these studies were not fully conclusive and the molecular mechanism(s) of pyrethroid resistance in *L*. *salmonis* await identification [22].

The aim of the current investigation was to obtain insights into the genetic basis of pyrethroid resistance in *L*. *salmonis*. To this end, crosses were performed between the drug susceptible *L*. *salmonis* strain IoA-00 and the pyrethroid resistant strain IoA-02, which displayed a ~100-fold difference in deltamethrin susceptibility, to produce families spanning one parental (P0) and three filial generations (F1-F3). Phenotyping the offspring of families derived from crosses between a resistant dam and a susceptible sire revealed maternal inheritance of deltamethrin resistance. This suggested the involvement of mitochondrial genetic factors in the resistance phenotype, paralleling a mechanism identified for bifenazate resistance in the phytophagous mite *Tetranychus urticae* Koch, 1836 [35,36], but unexpected for pyrethroids. To investigate the hypothesis of mitochondrial determinants of pyrethroid resistance in *L*. *salmonis* further, the mitochondrial genome was characterised for strains IoA-00 and IoA-02, as well as for four further strains of known deltamethrin susceptibility, isolated from salmon farming sites located in different regions of the UK, and for archived *L*. *salmonis* sampled from wild salmon in 2010. Moreover, the possibility that deltamethrin might have mitochondrial targets in *L*. *salmonis* was investigated by studying deltamethrin effects on total body ATP levels in IoA-00 and IoA-02 parasites.

## Results

### Design and performance of salmon louse crosses

In order to obtain insights into the genetic basis of pyrethroid resistance in *L*. *salmonis*, crosses were carried out between two laboratory-maintained strains of the parasite differing in drug susceptibility. While strain IoA-00 is susceptible to all licensed salmon delousing agents, strain IoA-02 is resistant to the pyrethroids deltamethrin and cypermethrin (Table 1). Gender differences in deltamethrin susceptibility were non-significant (p > 0.05) in both strains. Deltamethrin concentrations measured in selected bioassays were in the range of 68.3% to 133.3% of nominal concentrations (S1 Fig).

**Table 1:** Susceptibility of salmon louse strains to pyrethroids. Bioassays involved 30 min of exposure of parasites to deltamethrin or cypermethrin, followed by 24 h of recovery and rating of animals as normal or affected. Standard bioassays included at least five drug concentrations with duplicate batches of 10 animals (5 adult males and 5 preadult II females) per drug or control treatment. Median effective concentrations (EC_50_) were derived by probit analysis. Single-dose bioassays were conducted where availability of parasites was restricted and involved one deltamethrin concentration (2 μg L^-1^) and untreated controls.

		EC_50_ (μg L^-1^)^a^ and 95% confidence limits	
Salmon louse strain	Origin^b^	Deltamethrin	Cypermethrin
IoA-00	Firth of Clyde, 2003	0.28 (0.23–0.36)	0.46 (0.27–0.79)
IoA-01	Sutherland, 2008	0.36 (0.26–0.46)	ND^c^
IoA-02	Shetland Islands, 2011	40.1 (22.1–158.9)	> 20^d^
IoA-03	Sutherland, 2012	> 2.0^e^	ND
F3 (P0: IoA-00 dam)	Crosses of this study^f^	0.26 (0.19–0.33)	ND
F3 (P0: IoA-02 dam)	Crosses of this study^f^	9.66 (5.81–23.5)	ND
NA01-O	Argyll and Bute, 2012	24.8 (12.2–85.7)	ND
NA01-P	Argyll and Bute, 2012	80.5 (32.5–614)	ND

^a^ Raw data used to derive EC_50_ are provided in S1 Table.

^b^ The geographical origin of isolates is shown in S2 Fig.

^c^ Not determined.

^d^ Highest drug concentration tested in a standard bioassay, at which less than 50% of parasites were affected.

^e^ In a single-dose bioassay, 93.2% of parasites (41 of a total of 44 animals) remained unaffected after exposure to 2.0 μg L^-1^ deltamethrin.

^f^ See text for details.

**Fig 1:**
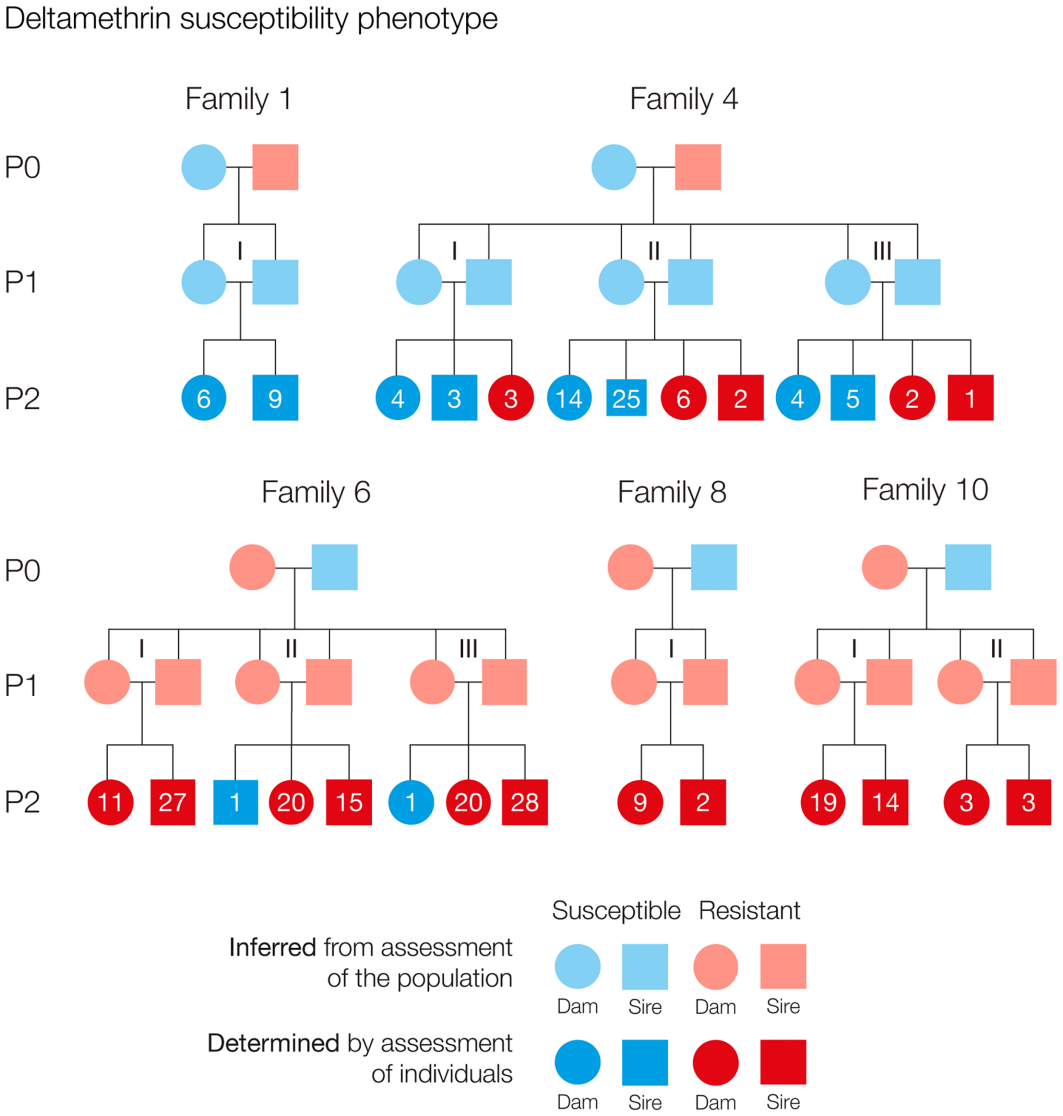
Pedigree chart of *L*. *salmonis* crosses. Families were initiated at parental generation P0 by setting up breeding pairs consisting of one parent from strain IoA-00 (drug susceptible) and one from strain IoA-02 (deltamethrin resistant). In each family, four sibling crosses between F1 animals were initially set-up, but not all were fertile. Within families, F1 pairs and their offspring are considered subfamilies and labelled by Roman numerals. The deltamethrin susceptibility of P0 parasites was inferred from bioassays on the parental strains (Table 1), while that of F1 parasites of the families was inferred on the basis of results of bioassays carried out on spare F1 siblings not required to propagate the cross (S3 Fig). The deltamethrin susceptibility of F2 animals was determined using single-dose bioassays involving exposure of salmon lice to 2 μg L^-1^ deltamethrin (30 min) and followed by recovery in seawater (24 h) before parasite behaviour was examined and rated. F2 animals were considered deltamethrin resistant when rated normal and susceptible to deltamethrin when rated affected. In the chart, numbers within symbols indicate the number of susceptible and resistant F2 males and females in each subfamily.

Crossing experiments were designed to produce fully pedigreed families spanning three generations, P0, F1 and F2, with both possible gender-strain orientation being represented at P0 (Fig 1). Families are identified by Arabic numerals. A significant number of families were discontinued because of a lack of egg production or loss of one or both breeding P0 parasites. To compensate against further losses, multiple crosses between F1 siblings were set up in each family. F1 sibling crosses within one family are referred to as subfamilies distinguished by Roman numerals added to the family identifier. F2 animals obtained from the first set of eggs produced by F1 females were sampled for genetic analyses after their deltamethrin susceptibility phenotype had been determined (see below).

### Pattern of inheritance of deltamethrin susceptibility

The deltamethrin susceptibility phenotype of F2 animals was determined by examination of parasite behaviour after 30 min of exposure to 2 μg L^-1^ deltamethrin and 24 h of recovery in seawater. Animals were considered resistant when rated normal and susceptible when rated affected (see Materials and methods for criteria). P0 and F1 animals were not subjected to deltamethrin exposures in order to not compromise reproduction. The phenotype of P0 animals was inferred from the deltamethrin susceptibility of their strains of origin, with IoA-00 animals being considered susceptible and IoA-02 parasites considered resistant. To assess the deltamethrin susceptibility phenotype of F1 animals, F1 individuals not required to propagate crosses were pooled within families of the same gender-strain orientations at P0 and subjected to deltamethrin bioassays. F1 animals descended from an IoA-00 sire and an IoA-02 dam were highly resistant to deltamethrin (EC_50_ > 10 μg L^-1^; S3 Fig). Due to a limited number of offspring being available, F1 parasites of families of the inverse orientation (P0: IoA-02 sire x IoA-00 dam) could be tested at only three drug concentrations. Results indicated that these F1 parasites were not deltamethrin resistant, with 90% of animals being immobilised after exposure to 2.5 μg L^-1^ deltamethrin (S3 Fig).

As observed in F1 parasites, the deltamethrin susceptibility of F2 parasites depended on the gender-strain orientation the P0 cross of the respective family. In families 1 and 4, which were derived from an IoA-00 dam and an IoA-02 sire, most F2 parasites were deltamethrin susceptible (83.3%, n = 84). In contrast, F2 animals from families 6, 8 and 10, which showed the inverse gender-strain orientation at P0, were almost all deltamethrin resistant (98.8%, n = 173) (Fig 1).

In order to investigate deltamethrin susceptibility in a further generation F3 of the cross, additional F2 siblings derived from a second clutch of eggs available in some F1 females were allowed to reproduce within families, with individual parenthood no longer being distinguished. F3 parasites from families of the same gender-strain orientation at P0 were pooled and subjected to deltamethrin bioassays. F3 parasites derived from subfamilies 4-II and 4-III, called ‘F3 (P0: IoA-00 dam)’, were deltamethrin susceptible (Table 1). In contrast, F3 animals originating from subfamilies 6-I, 6-III and 10-III, called ‘F3 (P0: IoA-02 dam)’, were deltamethrin resistant (Table 1).

### Mitochondrial genome assembly

The above observations demonstrated maternal inheritance of deltamethrin resistance in families descended from a resistant P0 dam, suggesting potential roles of mitochondrial genes in the resistance phenotype. In order to support further investigations into such potential roles of mitochondrial DNA (mtDNA), the *L*. *salmonis* mitochondrial genome was studied in selected P0 and F2 animals from the above crosses. In addition, mtDNA sequences were obtained from parasites of one further deltamethrin susceptible and three further deltamethrin resistant strains, established from isolates collected at farm sites in different UK regions (Table 1), as well as from parasites collected in 2010 from wild host fish caught at a salmon river located on the East coast of Scotland, a region lacking salmon farms. Five overlapping amplicons covering the entire *L*. *salmonis* mitochondrial genome were generated by PCR (S2 Table) and sequenced on both strands (S3 Table), followed by sequence assembly. A total of 1,212 sequences were produced for 35 samples (S4 Table).

**Fig 2:**
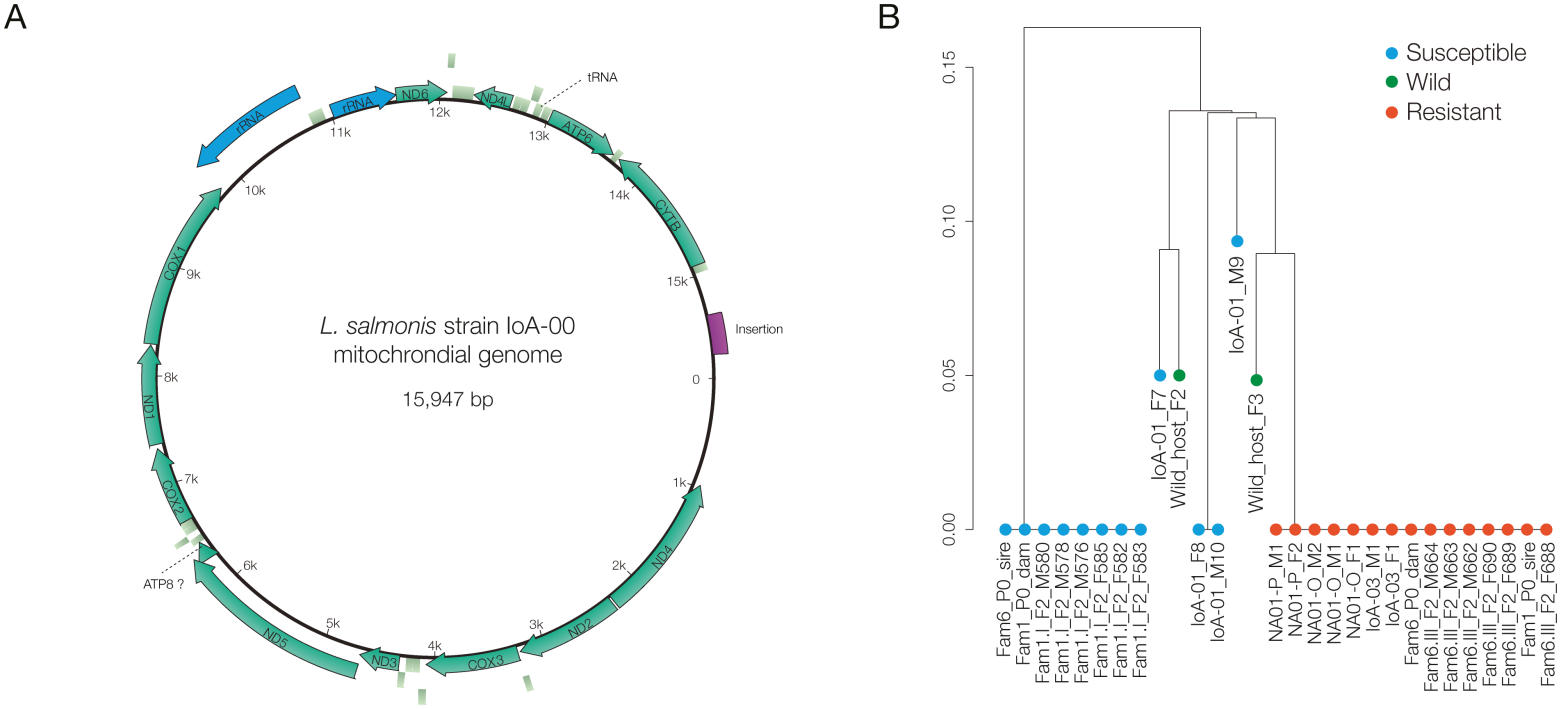
Analysis of the mitochondrial genome in *L*. *salmonis*. A, The reference IoA-00 mitochondrial genome has a size of 15,947-nt and comprises 13 protein coding genes (green arrows), 22 tRNA (light green rectangles) and 2 rRNA (blue arrows), The long insert in the D-Loop region (compare to NC_007215.1) is represented in purple. B, Phylogenetic tree built from individual mitochondrial genomes of *L*. *salmonis* of different origin (arbitrary scale produced by identity-by-state analysis). Please see text and Table 1 for more information on the *L*. *salmonis* populations included. The pedigree of *L*. *salmonis* individuals derived from crosses is provided in S5 Table. Please see Fig 1 for an outline of crosses.

A *L*. *salmonis* reference mitochondrial genome was constructed based on sequences obtained from eight deltamethrin-susceptible parasites from the above crosses, namely the IoA-00 P0 dam of family 1, the IoA-00 P0 sire of family 6 and six F2 individuals of subfamily 1-I. The obtained reference IoA-00 mitochondrial genome is 15,947 nt long, and exhibits 13 protein coding genes (cytochrome *B*, ATPase subunits 6 and 8, NADH dehydrogenase subunits 1–6 and 4L, cytochrome *c* oxidase subunits I-III), 22 tRNA and 2 rRNA (Fig 2A). The same protein coding genes have been found in a previously published assembly with identical organisation on the mitochondrial chromosome [37]. Compared to the assembly published by Tjensvoll and co-workers [37], the IoA-00 mitochondrial genome shows 160 single nucleotide polymorphisms (SNP), 5 single nucleotide deletions, 8 insertions and one small 23-nt deletion and one large insert of 528 nt (Fig 2A).

### Phylogenetic analysis of mitochondrial genomes

After the assembly and alignment of mitochondrial genomes of individual parasites analysed in this study, a phylogeny was constructed (Fig 2B). As expected, the F2 parasites of two subfamilies analysed each shared identical sequences with the P0 dam of their respective family, and thus assigned to two clusters in the obtained tree, confirming complete maternal inheritance of mtDNA in *L*. *salmonis* (Fig 2B). Moreover, all analysed deltamethrin resistant parasites, i.e. animals from strains IoA-02, IoA-03, NA01-O and NA01-P as well as F2 parasites from subfamily 6-III, had virtually identical mtDNA sequences, with only one nucleotide difference in the entire mitochondrial genome, and clustered together in the phylogenetic analysis (Fig 2B). In contrast, mtDNA sequences varied among the deltamethrin susceptible individuals analysed, consisting of parasites of strains IoA-00 and IoA-01, as well as F2 *L*. *salmonis* of family 1 (Fig 2B). In the phylogenetic tree, sequences of *L*. *salmonis* sampled from wild host fish grouped closer to the cluster containing IoA-02 than to the one containing IoA-00 parasites (Fig 2B).

### SNP association

Among the 35 individuals subjected to mtDNA analyses, the entire mitochondrial genome was sequenced for 27 parasites, while in the remaining 8 animals analyses were restricted to selected highly polymorphic mtDNA regions. Nine haplotypes were identified from *L*. *salmonis* individuals analysed, of which six were found in parasites with known phenotype (Table 2) and three in sea lice collected from wild hosts. All deltamethrin resistant individuals possessed haplotypes A or A*, which differ by only one SNP. Haplotypes A/A* differ from all other haplotypes by only 28 variations (Table 3). None of these sequence variations were predicted to result in truncated proteins or disrupt the expression of any proteins or RNAs. Ten of 13 protein coding genes showed SNPs, with non-synonymous mutations evident in the genes encoding NADH dehydrogenase subunits 1 and 5, and cytochrome *c* oxidase subunit III (Table 3).

**Table 2:** Mitochondrial haplotypes in *L*. *salmonis* analysed.

Sample^1^	Sex	Phenotype^2^	Haplotype^3^	Coverage	Genome type
Fam1_P0_sire	M	Resistant	A	100%	Complete Genome
Fam6-III_P0_dam	F	Resistant	A	100%	Complete Genome
Fam6-III_F2_F688	F	Resistant	A	100%	Complete Genome
Fam6-III _F2_F689	F	Resistant	A	100%	Complete Genome
Fam6-III _F2_F690	F	Resistant	A	100%	Complete Genome
Fam6-III _F2_M662	M	Resistant	A	100%	Complete Genome
Fam6-III _F2_M663	M	Resistant	A	100%	Complete Genome
Fam6-III _F2_M664	M	Resistant	A	100%	Complete Genome
IoA-03_F1	F	Resistant	A or A*	16%	Genome Fragment
IoA-03_M1	M	Resistant	A or A*	16%	Genome Fragment
NA01-P_F2	F	Resistant	A*	75%	Complete Genome
NA01-P _M1	M	Resistant	A*	75%	Complete Genome
NA01-O_F1	F	Resistant	A*	75%	Complete Genome
NA01-O _M1	M	Resistant	A*	75%	Complete Genome
NA01-O _M2	M	Resistant	A*	75%	Complete Genome
Fam1_P0_dam	F	Susceptible	B	100%	Complete Genome
Fam1-I_F2_F582	F	Susceptible	B	100%	Complete Genome
Fam1-I_F2_F583	F	Susceptible	B	100%	Complete Genome
Fam1-I_F2_F585	F	Susceptible	B	100%	Complete Genome
Fam1-I_F2_M576	M	Susceptible	B	100%	Complete Genome
Fam1-I_F2_M578	M	Susceptible	B	100%	Complete Genome
Fam1-I_F2_M580	M	Susceptible	B	100%	Complete Genome
Fam6_P0_sire	M	Susceptible	B	100%	Complete Genome
IoA-01_M9	M	Susceptible	C	100%	Complete Genome
IoA-01_F1	F	Susceptible	C	37%	Genome Fragment
IoA-01_M2	M	Susceptible	C	37%	Genome Fragment
IoA-01_F3	F	Susceptible	C	37%	Genome Fragment
IoA-01_M5	M	Susceptible	C	37%	Genome Fragment
IoA-01_F7	F	Susceptible	D	100%	Complete Genome
IoA-01_F8	F	Susceptible	E	100%	Complete Genome
IoA-01_M10	M	Susceptible	E	100%	Complete Genome

^1^ The pedigree of individuals derived from crosses that were subjected to mtDNA sequencing is provided in S5 Table.

^2^Deltamethrin susceptibility phenotype. Please refer to Table 1 and Fig 1 for details

^3^ Haplotype A* same as haplotype A except for one single SNP (position 203 A -> G). Haplotype B: reference IoA-00 mitochondrial genome.

**Table 3:** Sequence variation between deltamethrin resistant and susceptible *L*. *salmonis*.

Position*	Type	Location	Description	Original*	Replaced by
714	Polymorphism	Intergenic		A	G
945	Polymorphism	Intergenic		G	A
1174	Synonymous	ND4	CTC/Leu -> CTT/Leu	G	A
1678	Synonymous	ND4	GAG/Glu -> GAA/Glu	C	T
3056	Synonymous	ND2	CTC/Leu -> CTT/Leu	C	T
3338	Non-Synonymous	COX3	GGG/Gly -> GAG/Glu	G	A
3348	Synonymous	COX3	GCT/Ala -> GCC/Ala	T	C
4563	Synonymous	ND3	AGG/Ser -> AGA/Ser	G	A
4787	Synonymous	ND5	CTA/Leu -> TTA/Leu	C	T
5889	Non-Synonymous	ND5	TTA/Leu -> TCA/Ser	T	C
6325	Synonymous	ND5	GTG/Val -> GTA/Val	G	A
6942	Synonymous	COX2	GGG/Gly -> GGA/Gly	G	A
8013	Synonymous	ND1	GGT/Gly -> GGC/Gly	T	C
8134	Non-Synonymous	ND1	GGG/Gly -> AGG/Ser	G	A
8600	Non-Synonymous	COX1	TTG/Leu -> TCG/Ser	T	C
8661	Synonymous	COX1	TAC/Tyr -> TAT/Tyr	C	T
9030	Synonymous	COX1	GGA/Gly -> GGG/Gly	A	G
9126	Synonymous	COX1	GGA/Gly -> GGG/Gly	A	G
9426	Synonymous	COX1	GGA/Gly -> GGG/Gly	A	G
10094	Polymorphism	l-rRNA		G	A
10722	Insertion	l-rRNA		T	TAG
11190	Polymorphism	s-rRNA		C	T
13466	Synonymous	ATP6	AGG/Ser -> AGA/Ser	G	A
14013	Synonymous	CYTB	TTT/Phe ->TTC/Phe	A	G
14061	Synonymous	CYTB	TTG/Leu -> TTA/Leu	C	T
14751	Synonymous	CYTB	GAG/Glu -> GAA/Glu	C	T
15381	Polymorphism	Intergenic		T	C
15466	Polymorphism	Intergenic		A	G

* Refers to IoA-00 Strain mitochondrial genome sequence.

### Drug effects on *L*. *salmonis* total ATP levels

**Fig 3:**
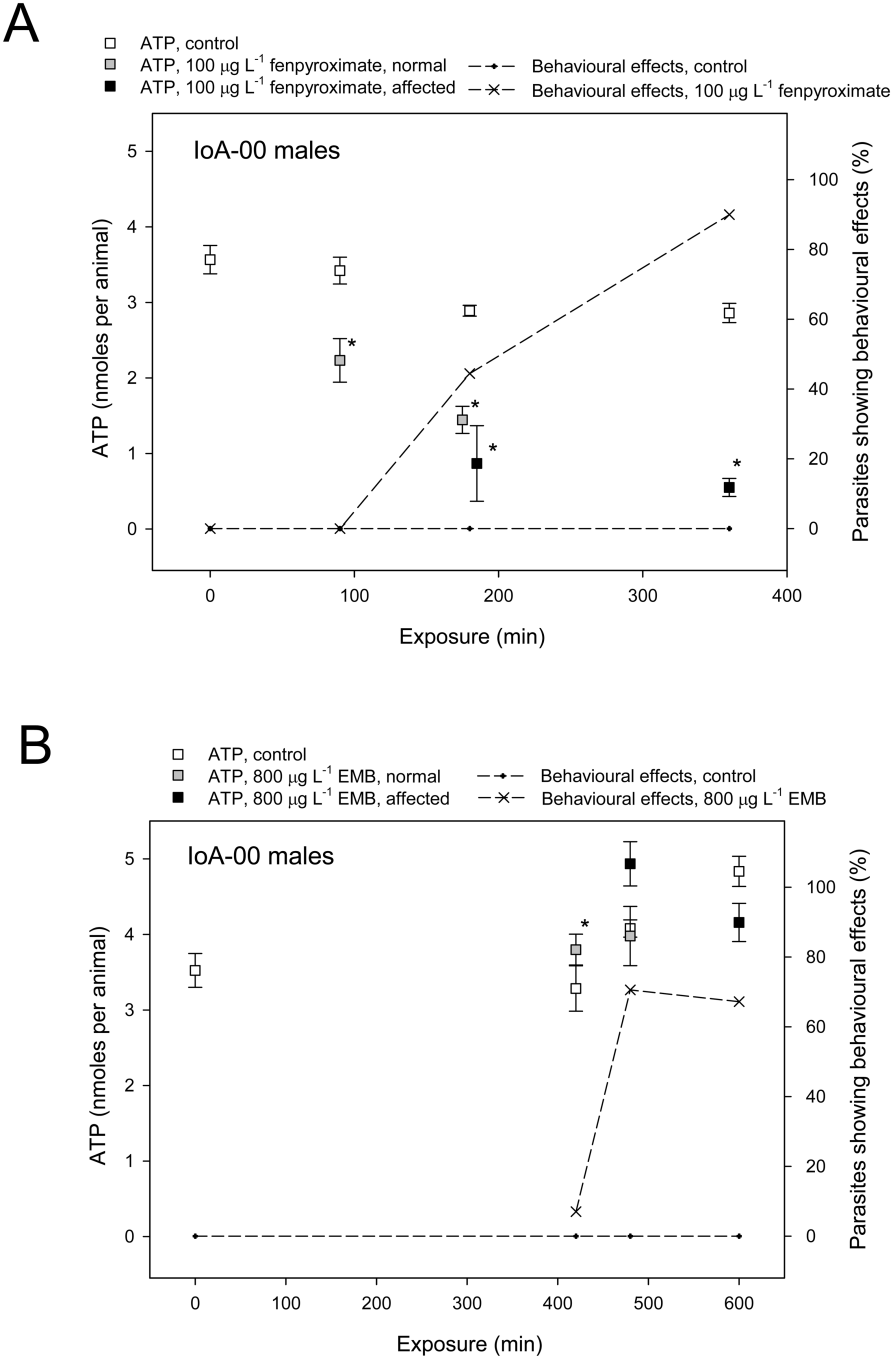
Effects of fenpyroximate and emamectin benzoate on ATP levels in *L*. *salmonis*. Male adult *L*. *salmonis* of strain IoA-00 were exposed to drugs or solvent PEG_300_ (0.05% v/v, controls) for different time periods before behavioural effects were recorded and animals were sampled followed by immediate homogenisation in phenol and storage at -20°C pending extraction and analysis of whole body ATP. Symbols represent average behavioural responses (n = 10–20) or the average and standard error of ATP levels (n = 4–12). Symbols indicate that ATP levels in a given group differed significantly from those found in the time-matched control group (p < 0.05, Dunnett’s test). A. Effects of fenpyroximate (100 μg L^-1^). B. Effects of emamectin benzoate (800 μg L^-1^).

**Fig 4:**
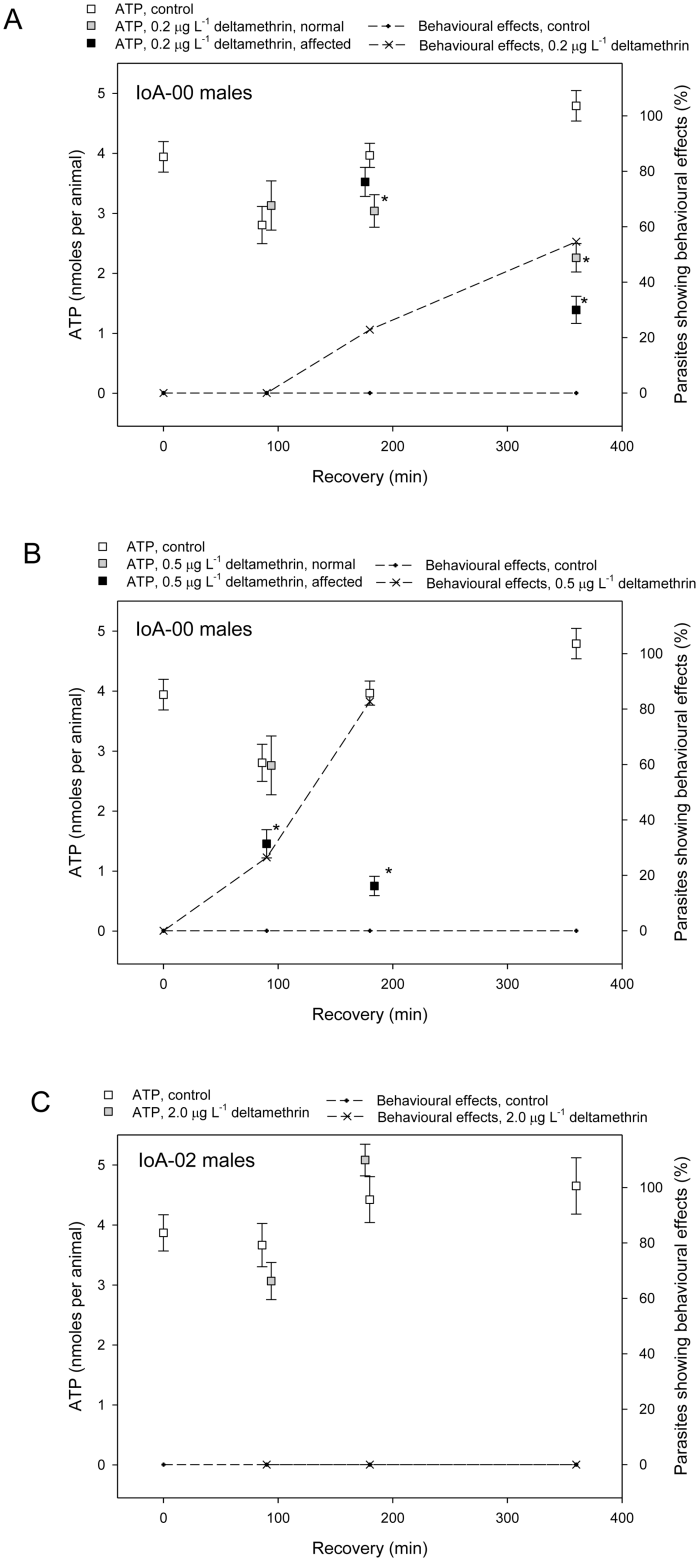
Effects of deltamethrin on ATP levels in *L*. *salmonis*. Male adult *L*. *salmonis* were exposed to deltamethrin or solvent PEG_300_ (0.05% v/v, controls) for 30 min and then allowed to recover in seawater. At different time periods during recovery, behavioural effects were recorded and animals were sampled for determination of whole body levels of ATP. See the legend of Fig. 4 for more details and explanation of symbols. A, Effects of 0.2 μg L^-1^ deltamethrin on IoA-00 parasites. B, Effects of 0.5 μg L^-1^ deltamethrin on IoA-00 parasites. C. Effects of 2.0 μg L^-1^ deltamethrin on IoA-02 parasites.

The maternal inheritance of deltamethrin resistance in families descended from a resistant P0 dam and the presence of two virtually identical mitochondrial haplotypes specific to all deltamethrin resistant parasites analysed suggested a contribution of mitochondrial genes to the resistance phenotype. These findings potentially imply mitochondrial targets of deltamethrin toxicity in *L*. *salmonis*. To investigate whether deltamethrin or other pesticides interfere with mitochondrial ATP production, their effects on whole body ATP levels were studied. Exposure of IoA-00 *L*. *salmonis* to 100 μg L^-1^ fenpyroximate, an acaricide that blocks oxidative phosphorylation, provoked behavioural toxicity in IoA-00 parasites within 6 hours and simultaneously depleted ATP levels (Fig 3A). In contrast, exposure of IoA-00 lice to 800 μg L^-1^ emamectin benzoate, an avermectin not known to interact with mitochondrial targets, caused toxic effects in ~70% of IoA-00 parasites within 10 hours but did not affect ATP levels (Fig 3B). When IoA-00 parasites were exposed to 0.2 μg L^-1^ or 0.5 μg L^-1^ deltamethrin for 30 min and monitored during recovery in clean seawater, toxic effects as well as decreases in ATP levels were observed within 6 h (Fig 4A & 4B). In contrast, no effects on toxicity or ATP levels were observed following the exposure of IoA-02 parasites to 2.0 μg L^-1^ deltamethrin (Fig 4C).

## Discussion

In reciprocal crosses between a deltamethrin-resistant and a drug-susceptible strain of *L*. *salmonis*, maternal inheritance of resistance was observed in families derived from crosses between resistant dams and susceptible sires. In these families, virtually all F2 individuals were resistant, as were 81.2% of F3 parasites. In contrast, in families descended from a susceptible dam and a resistant sire, less than 20% of F2 parasites were resistant while F3 parasites were susceptible.

The maternal inheritance of resistance in families descended from a resistant dam is unlikely to be mediated by chromosomal genes. While maternal inheritance can be based on the transfer of gene products from nurse cells to the egg (‘maternal effect’) or on epigenetic effects (‘genomic imprinting’), such effects would usually not extend to the F3 generation [38]. Genes located on sex chromosomes can also be excluded as potential determinants for the observed maternal inheritance, as regardless of sex determination system (likely ZW/ZZ in *L*. *salmonis*, [39]), segregation of resistance alleles should lead to the emergence of significant numbers of susceptible animals in the F2 generation of families. A more plausible explanation of the maternal inheritance of deltamethrin resistance in families derived from a resistant dam was that the maternally transmitted mitochondrial genome adopts potential roles in the resistance phenotype [40].

Conversely, the occurrence of resistant F2 parasites in one of two families descended from crosses between a resistant sire and a susceptible dam could be explained by a contribution to the resistance phenotype by nuclear gene(s) for which resistance alleles are not fixed in the resistant parental strain IoA-02. A role of nuclear genes in the resistance phenotype is further suggested by the less pronounced deltamethrin resistance of F3 parasites descended from a resistant dam as compared to strains IoA-02, NA01-O and NA01-P, despite all of these parasites sharing virtually identical mitochondrial haplotypes. The limited number of resistant F2 parasites in these families precluded elucidating the nature of potential nuclear genetic determinants of resistance. Further studies are required to determine the identity and mode of inheritance of these hypothetical nuclear determinants.

Based on the above results, the present study focused on investigating potential mitochondrial determinants of deltamethrin resistance. In a previous study the mitochondrial genome has been found to be highly polymorphic within different *L*. *salmonis* samples obtained from farmed and wild hosts of different geographical areas, with little evidence of genetic differentiation between sites [41]. In contrast, almost identical mitochondrial haplotypes were found in the deltamethrin resistant strain IoA-02, derived from an isolate collected in the Shetland Islands, as well as three further unrelated deltamethrin-resistant *L*. *salmonis* strains originating from other Scottish areas (Argyll and Bute, Sutherland). Compared to mitochondrial haplotypes from non-resistant parasites, deltamethrin resistance-associated haplotypes differ at 28 SNPs, with ten of 13 mitochondrial genes showing resistance-associated sequence polymorphisms. Independent research conducted in parallel with the current study provides further evidence for a maternal inheritance of deltamethrin resistance from reciprocal crosses between deltamethrin resistant and drug susceptible *L*. *salmonis* [42]. After sequencing two mitochondrial genes, five SNPs associated to resistance were identified, two of which are identical to SNPs identified in this study (positions 8600 and 9030) [42]. Taken together, the maternal inheritance of deltamethrin resistance and the lack of mtDNA sequence variability in deltamethrin resistant isolates suggest the clonal expansion of a mitochondrial haplotype associated with increased fitness during pyrethroid treatment, driven by the high potential of the parasite for dispersal and ubiquitous selection by pyrethroids on farmed sites in different salmon producing countries of the North Atlantic. While the available results do not allow excluding the possibility that the spread of this mitochondrial haplotype is secondary to the primary effect of another maternally transmitted factor, e.g. an endosymbiont or pathogen [40], the most parsimonious explanation is that mitochondrial genetic factors contribute directly to the resistance phenotype. This would suggest that mechanisms of pyrethroid resistance, and by inference potentially also the mechanism of pyrethroid toxicity, differ between *L*. *salmonis* and terrestrial arthropods, in which pyrethroid resistance is based on target site and metabolic mechanisms not involving the mitochondrial genome.

In insects, pyrethroid toxicity is based on drug binding and blockage of the neuronal voltage gated sodium channels, Na_V_, resulting in the disruption of neurotransmission [11]. *Kdr* (knockdown) resistance against pyrethroids is based on target site mutations of Na_V_, preventing pesticide effects on channel gating [14,43]. Three genes showing high homology to insect Na_V_ channels have been identified in *L*. *salmonis* [44]. A cDNA comprising a full coding sequence has been isolated for *L*. *salmonis* Na_V_1.1 [34,44], whereas partial sequences have been obtained for Na_V_1.2 and Na_V_1.3 [44]. No putative *kdr* type mutations of Na_V_1.1 could be identified after sequences homologous to Na_V_ regions harbouring *kdr* mutations had been amplified by PCR and sequenced in both drug-susceptible and pyrethroid resistant *L*. *salmonis* [44]. While further experiments considering Na_V_1.2 and Na_V_1.3 are required before definite conclusions can be drawn, attempts to detect *kdr* type mutations in pyrethroid resistant *L*. *salmonis* isolates have so far been unsuccessful [44].

In view of the maternal inheritance of pyrethroid resistance and its association with mitochondrial haplotypes, it is noteworthy that deltamethrin caused ATP depletion in drug susceptible *L*. *salmonis*. Effects were found with deltamethrin levels close to the EC_50_ of the drug and at early time points after exposure, coinciding with the first onset of behavioural toxic effects. In contrast, ATP levels remained unchanged after exposure to the avermectin emamectin benzoate at a concentration causing behavioural toxicity. It is tempting to speculate that deltamethrin effects on ATP levels might be related to a specific interaction of the drug or its metabolites with oxidative phosphorylation. However, more research is needed to confirm or refute this possibility. Based on the available results, it cannot be excluded that the depletion of ATP levels observed in this study may represent a secondary effect arising from deltamethrin toxicity.

While it is generally accepted that pyrethroids act mainly through interaction with Na_V_, there is evidence for the interaction of pyrethroids with other molecular targets. However, at least in mammals, concentrations required to provoke pharmacological effects through such non-classical targets are 2–3 orders of magnitude greater than those required to alter Na_V_ channel gating [45]. A number of studies have reported pyrethroid effects on mitochondrial function. While permethrin and cyhalothrin inhibited the activity of respiratory complex I *in vitro* in rat liver mitochondria [46], deltamethrin was found to inhibit oxygen consumption of isolated rat liver mitochondria and alter inner mitochondrial membrane potential [47]. After analysis of the respiratory chain, the localisation of the deltamethrin inhibition site between complexes II and III was suggested [47]. Interestingly, deltamethrin resistant *L*. *salmonis* investigated in this study showed non-synonymous mutations in the genes coding for NADH dehydrogenase subunits 1 and 5, which contribute to respiratory complex I, and cytochrome *c* oxidase subunit III, which is part of respiratory complex IV. In rodent models, deltamethrin further affected mitochondrial functions other than respiration. In particular, deltamethrin activated mitochondrial apoptotic pathways in rat brain cells [48] and disrupted steroid biosynthesis in mouse Leydig cells by causing mitochondrial membrane damage [49].

In addition to *kdr* discussed above, metabolic resistance is a further common mechanism of pyrethroid resistance in terrestrial arthropods and involves the enhanced expression of biochemical detoxification pathways [2]. The two main routes of metabolism of pyrethroids are hydroxylation at different positions by cytochrome P450 monooxygenases (CYPs) and hydrolysis of the central ester bond by esterases or other degradative enzymes [8,50]. The relative importance of these two metabolic routes shows interspecific variability in both insects and mammals [51,52]. CYP genes constitute a large gene family with roles in many physiological processes, including resistance to different pesticide classes [53]. The number of CYP family members in *L*. *salmonis* is unknown and only few studies have investigated roles of CYPs in caligid sea lice. The CYP inhibitor piperonyl butoxide increased cypermethrin and deltamethrin toxicity in *L*. *salmonis*, suggesting the involvement of CYPs in pyrethroid detoxification [33]. In the caligid species *C*. *rogercresseyi*, semi-quantitative RT-PCR revealed the induction of CYP3A27 and inhibition of CYP2M1 transcription by emamectin benzoate exposure [54]. In contrast, exposure to emamectin benzoate had little effect on transcript profiles for *L*. *salmonis* in two independent microarray studies that both failed to find clear transcript regulation of CYPs [55,56].

Compared to resistance based on enhanced CYP expression [2,18], resistance based on the overexpression of esterases [57] is less common in insects. In addition to esterases, certain serine proteases are capable of hydrolysing pyrethroids and have been found to be overexpressed in pyrethroid resistant insects [58,59]. Caligid sea lice are known to express significant levels of proteases that are involved in a number of key physiological processes including yolk utilisation in larvae, attachment to the host during infection, and digestion of the host’s skin tissues [60,61]. Interestingly, 44 putative trypsin-like and 7 putative chymotrypsin-like transcripts were identified in *C*. *rogercresseyi*, of which 5 showed upregulation in response to deltamethrin or azamethiphos treatment [62]. In an earlier study investigating *L*. *salmonis* transcript profiles using microarray analysis, transcripts of a metalloprotease were ~15-fold increased in emamectin benzoate resistant strain IoA-01 compared to susceptible strain IoA-00 in the absence of drug exposure [55]. Similarly, in a study investigating emamectin benzoate resistant and susceptible *L*. *salmonis* isolates from the east coast of Canada, higher constitutive transcript abundances for a number of degradative enzymes, including collagenase, were found in resistant animals [56]. Taken together, while a number of studies point to potential roles of biotransformation enzymes and degradative enzymes in drug metabolism in sea lice, more research is required to elucidate the specific pathways of deltamethrin metabolism in *L*. *salmonis* and unravel the roles of enzymes involved in drug biotransformation in pyrethroid resistance.

## Materials and methods

### Ethics statement

All research projects involving the Institute of Aquaculture (IoA) are subjected to a thorough Ethical Review Process prior to any work being approved. All projects with IoA participation are required to be submitted to the IoA Ethical Committee for approval, irrespective of where experimentation will be carried out. The forms to be completed for the ethical review process require all aspects of the experimentation to be described including conditions for the human experimenters as well as animal subjects. This procedure ensures all ethical issues are addressed before an experiment can be initiated. The present research was assessed by the IoA Ethical Review Committee and passed the Ethical Review Process of the University of Stirling (Project ID ASPA10/2013). Laboratory infections of Atlantic salmon with *L*. *salmonis* were carried out under UK Home Office project license PPL 60/4522.

### Lepeophtheirus salmonis

Different *L*. *salmonis* laboratory-maintained strains were used in this study, all of which were established from egg strings obtained from female parasites collected at different Scottish commercial salmon production sites (S2 Fig). Strain IoA-00 was taken into culture in 2003 and originated from a farm site in the Firth of Clyde system where no chemical control agents other than hydrogen peroxide had been used [63]. IoA-00 is susceptible to all current anti-sea louse agents including deltamethrin (Table 1). Strain IoA-01, which originates from Sutherland and has been in culture from 2008, shows resistance to emamectin benzoate [55] but is susceptible to deltamethrin (Table 1). Strain IoA-02 was established in 2011 from an isolate collected in the Shetland Islands and is resistant to deltamethrin, cypermethrin (Table 1) and emamectin benzoate [64]. IoA-03 (Sutherland), NA01-O and NA01-P (Argyll and Bute) are further deltamethrin resistant strains established in 2012 (Table 1). Under culture, strains have been maintained under identical conditions using *S*. *salar* as the host species, as described in detail elsewhere [63]. In addition to the laboratory strains, adult female *L*. *salmonis* collected from wild Atlantic salmon caught in 2010 in the river Esk, Lothian, East Coast of Scotland were also used for genetic analysis in this study.

### *L*. *salmonis* crosses

Strains IoA-00 and IoA-02 were crossed to produce families spanning four generations, respectively termed P0, F1, F2 and F3. Each family was established at the P0 level by adding one *L*. *salmonis* pair to a tank containing one Atlantic salmon smolt (~200 g). In five of ten families, the P0 pair consisted of an IoA-00 adult male (P0 sire) and an IoA-02 preadult-II female (P0 dam), whereas in the remaining families the P0 pair had the inverse gender-strain orientation. Prior to the set-up of crosses, the virginity of P0 dams was ascertained by examining the genital segment for the presence of stored spermatophores under a microscope. After the introduction of P0 pairs to tanks, females were examined twice a week for signs of insemination. Once successful mating had been confirmed, the P0 sire was recovered and stored in absolute ethanol pending DNA extraction. P0 dams were maintained to produce egg strings, which were removed and incubated to allow hatching and early larval development [65]. The resulting infective F1 copepodid larvae were then used to inoculate tanks containing naïve Atlantic salmon (~500 g, one tank with ten fish per family). After successful settlement of F1 parasites on host fish, P0 dams were recovered into absolute ethanol while removing the genital segment to preclude contamination by male DNA from stored sperm. Infections were maintained until F1 parasites reached the adult male and preadult-II female stages. At this point, five of ten families initially set up had been lost due to natural mortality or failure to produce progeny. To compensate for similar losses in the next generation, four breeding sibling pairs of F1 parasites, referred from here on as subfamilies, were set up per family as described above and maintained to produce F2 egg strings. Available F1 parasites not required for propagation of the cross were subjected to deltamethrin bioassays (see below). The first two sets of F2 egg strings produced by F1 females were sampled and allowed to develop into infective copepodids, which were used to inoculate separate batches of naïve host fish, employing the same experimental steps as used for F1 infections. F1 sires and dams were sampled after confirmation of insemination or successful F2 infections, respectively. F2 parasites obtained from the first set of egg strings were maintained until reaching the male adult / female preadult II stages, at which point their deltamethrin susceptibility phenotype was assessed by bioassay (see below), followed by sampling of animals for DNA analyses. At this point in the experiment, results obtained with F1 and F2 parasites had revealed a predominantly maternal inheritance of deltamethrin resistance. To allow further characterisation of the deltamethrin susceptibility phenotype in lines derived from P0 crosses having opposite gender-strain orientations (IoA-00 sire x IoA-02 dam or inverse), a third filial generation F3 was made by crossing F2 animals, using the second batch of F2 parasites produced. Individual parentage was no longer recorded in these crosses, which were performed by maintaining F2 infections up to the time point where F2 siblings had mated naturally and females produced egg strings. Egg strings were collected and pooled within subfamilies derived from P0 crosses of the same gender-strain orientation (IoA-00 sire x IoA-02 dam or inverse). F3 parasites were produced in the same manner as outlined above, involving two experimental infections (one per gender-strain orientation at P0). When F3 parasites reached the adult male and preadult-II female stages, their deltamethrin susceptibility was characterised in bioassays (see below).

### *L*. *salmonis* bioassays

Adult male and preadult II female *L*. *salmonis* were collected from host fish that had been anaesthetised using 2-phenoxyethanol (100 mg L^-1^; 99%; Sigma-Aldrich, U.K.) or killed by a UK Home Office approved Schedule 1 method (exposure to overdose of ethyl 3-aminobenzoate methanesulfonate (100 mg L^-1^) followed by destruction of the brain). After collection, parasites were allowed to recover in aerated filtered seawater equilibrated to 12°C (from here on simply called seawater) for 2–4 h. During the set-up of bioassays, *L*. *salmonis* were randomly allocated to 300 mL crystallising dishes containing 100 mL of seawater, with each vessel receiving 5 males and 5 females. PEG_300_ (polyethylene glycol, M_n_ = 300) was used to solubilise pyethroids deltamethrin and cypermethrin (both Pestanal^®^ analytical standard, Sigma-Aldrich, U.K.). A stock solution of 0.5 mg mL^-1^ of the appropriate pyrethroid was prepared in PEG_300_ and diluted to produce for each planned exposure concentration, a matching 2000x final test-concentration solution in PEG_300_. Two types of salmon louse bioassays were conducted within this study, standard biossays [66] and single-dose bioassays [67]. Standard bioassays, used to characterise the drug susceptibility of salmon louse strains, included a geometrical series of at least five concentrations of the tested drug and a solvent control. Selected experiments further included a seawater control. Two replicate dishes, both containing five parasites of either sex, were used per drug or control treatment. Single-dose bioassays, which were used to characterise the drug susceptibility of individual F2 parasites from crosses or determine the susceptibility of salmon louse strains where availability of test animals was restricted, employed one diagnostic deltamethrin concentration (2 μg L^-1^) only, supplemented by an external solvent control. The final PEG_300_ concentration in deltamethrin treatments and solvent controls was 0.05% (v/v). Deltamethrin exposures were initiated by adding to the above crystallising dishes containing 100 mL of seawater and parasites, 100 mL of a 2x final-concentration deltamethrin solution that had been prepared immediately before by combining 100 μL of the appropriate 2000x final-concentration solution and 100 mL of seawater. Solvent controls received 100 mL of seawater containing 0.1% (v/v) PEG_300_, while controls received the same amount of seawater. After 30 min of exposure at 12°C, the exposure solutions were carefully decanted from dishes while retaining the parasites. Parasites were then rinsed twice with seawater and transferred to 150 mL plastic Petri dishes holding 70 mL of seawater. Animals were then allowed to recover for 24 h in an incubator set to 12°C before their attachment and motility behaviour was rated. During blind examination by an observer unaware of treatment history and strain affiliation of animals, gentle stimulation with a fine brush was applied while animals were inspected visually. Behavioural responses were rated according to criteria proposed by Igboeli and co-workers [68], which are slight modifications of earlier definitions [66,69]. Parasites were rated “live” when firmly attached to the walls of the dish or swimming normally, “weak” when swimming irregularly and failing to attach firmly (animals may attach briefly, but dislodge again instantly), “moribund” when incapable of swimming away or attaching (animals may twitch appendages or move uncoordinatedly in a close circle), and “dead” when showing no movements in extremities, gut or other organs as apparent from examination under a microscope. Parasites were considered normal when rated “live” or “weak” and affected when rated “moribund” or “dead”. Bioassays were considered invalid if more than 10% of parasites in control treatments were deemed affected.

### Deltamethrin residue analyses

Deltamethrin concentrations in exposure solutions of selected bioassays were analysed by a commercial laboratory (RPC Science and Engineering, Fredericton, NB, Canada). Water samples (50 mL) taken at the beginning and the end of salmon louse exposures were stored in borosilicate glass bottles and frozen before being shipped to the analytic laboratory on dry ice. Analytical procedures involved solvent extraction followed by gas chromatography and electron capture detection.

### Effects of deltamethrin on *L*. *salmonis* ATP levels

Exposure experiments performed to assess deltamethrin effects on whole body ATP levels followed essentially the same methodology as bioassays. Test animals were adult male parasites from synchronised cohorts of IoA-00 and IoA-02 strains. In addition to deltamethrin, two further drugs were studied. The acaricide fenpyroximate is known to act by interference with the mitochondrial complex I [70] and thus was expected to cause depletion of ATP levels. In contrast, specific effects on oxidative phosphorylation were not expected for the salmon delousing agent emamectin benzoate, an avermectin believed to act through interference with neuronal ligand-gated chloride channels [71]. Experiments assessed drug effects on ATP levels at early time points following exposure, timed to coincide with the first onset of apparent effects on behaviour. Trials with emamectin benzoate and fenpyroximate involved the continuous exposure of *L*. *salmonis* for 0 to 10 h. At set time points animals were sampled for ATP analyses after their behavioural responses had been rated according to categories given above. Trials with deltamethrin involved 30 min of drug exposure followed by 0–6 h recovery, during which animals were sampled after rating. Emamectin benzoate (800 μg L^-1^) and fenpyroximate (100 μg L^-1^) were tested in strain IoA-00. Deltamethrin was tested at 0.2 μg L^-1^ and 0.5 μg L^-1^ in strain IoA-00 and at 2.0 μg L^-1^ in strain IoA-02. Drugs were solubilised in PEG_300_ (final concentration: 0.05% v/v) as described above. Animals in control treatments were exposed to seawater containing 0.05% (v/v) PEG_300_. In experiments, four dishes with 10 parasites were set up for controls, allowing the sampling of ten animals each at time point zero and three further time points. In drug treatments, six dishes were set up to support the sampling of 20 parasites per time point, allowing separate assessment of ATP levels for animals rated as “normal” or “affected”.

The ATP content of adult male *L*. *salmonis* was quantified using a commercially available kit (A-22066, Molecular Probes, Thermo Fisher Scientific, USA). Directly after behavioural rating, each animal sampled was added to a plastic tube containing 1 mL of ice-cold phenol and glass beads and homogenised in a BeadBeater device (BioSpec, USA) using two 50 sec cycles. After addition of 200 μL chloroform and 150 μL distilled water to samples, tubes were thoroughly shaken for 20 sec and the homogenate centrifuged at 4°C at 25,000 *g* for 15 min. The resulting aqueous supernatant was diluted 1/10 and immediately used in luciferase assays. Luminescence activities were obtained using a Synergy 2 multi-mode plate reader (BioTek, UK). Plates included ATP standards to allow quantification of the ATP content in the samples.

### DNA extraction

Genomic DNA was extracted from individual *L*. *salmonis* using a DNA extraction buffer [72] (Final concentrations; 17.5 g mL^-1^ NaCl; Tris Base, 6.05 g mL^-1^; EDTA, 58.45 g L^-1^; EGTA, 76 g L^-1^; spermidine, 72 g L^-1^; spermine, 52 g L^-1^). For each adult or preadult parasite, 360 μL DNA extraction buffer, 40 μL 10% SDS, and 5 μL proteinase K (10 g L^-1^) was added. Samples were then incubated for 2 h at 55°C in a rotator set to low speed. After incubation, samples were heated for 15 min at 70°C to inactive proteinase K and then allowed to cool to room temperature. After addition of 5 μL of RNase A (2 g L^-1^) samples were incubated for 60 min at 37°C in a rotator set to a low speed. After the incubation, 290 μL of 5 M sodium chloride were added and samples were incubated on ice for 10 min to precipitate proteins. Following centrifugation at maximum speed (> 25,000 *g*) for 15 min, 300 μL of the obtained supernatant were collected. To precipitate the extracted DNA, 300 μL of isopropanol (room temperature) were added to the supernatant. Samples were mixed by inversion and incubated on ice for 5 min before the DNA was sedimented by centrifugation at maximum speed (> 25,000 *g*) for 15 min. The resulting supernatant was discarded, while the pellet was washed twice with 1 mL of 75% ethanol, involving incubations (first: 2 h, second: overnight) followed by centrifugation at high speed (> 21,000 *g*, 5 min) to pellet the DNA. Following decanting of the supernatant and removal of excess ethanol, samples were air-dried before DNA was re-suspended in 15 μL of 5 mM Tris buffer (pH 8.5).

### Mitochondrial genome (mtDNA) amplification

The full mitochondrial genome was amplified from six laboratory maintained strains of *L*. *salmonis* of known pyrethroid susceptibility (IoA-00, IoA-01, IoA-02, IoA-03, NA01-P and NA01-O; Table 1) and salmon lice collected from wild salmon captured on the East Coast of Scotland in 2010. Specific PCR oligonucleotide primers designed to target the *L*. *salmonis* mitochondrial genome [GenBank: NC_007215.1] were used to amplify the mtDNA in 5 overlapping products (S2 Table). Fifty nanograms of total DNA were used as PCR template with 0.3 μM of each oligonucleotide, 5 mM of each dNTP and 5 units of Takara LA Taq Hot Start polymerase (Takara Bio, USA) in a total volume of 40 μL. Reactions were run in a Mastercycler RealPlex (Biometra, UK). PCR conditions consisted of a denaturing step at 96°C for 2 min, followed by 30 cycles of denaturation at 96°C for 30 sec, annealing at the specified temperature (S2 Table) and extension at 72°C for 1 min/kb. PCR products were purified (QIAquick PCR Purification Kit, QIAGEN, USA) and sequenced in both directions (S3 Table) using capillary sequencing technology (Macrogen, Netherlands).

### Mitochondrial genome assembly

Each sequence generated was manually assessed and the trimming coordinates were recorded for tractability (S4 Table). The trimmed sequences were extracted and filtered using Trimmomatic (v0.36).

The sequences of deltamethrin susceptible individuals from the crosses analysed (two IoA-00 P0 parents, six F2 parasites of subfamily 1.1) were aligned to the *L*. *salmonis* mitochondrial reference genome (NC_007215.1) using bowtie2 v2.2.6 [73] in order to build a reference genome for the Scottish parasite populations studied here. A consensus sequence was built and annotations were transferred from NC_007215.1 using RATT release 18 [74]. Annotations of tRNA were added using MITOS release 806 [75]. RATT was edited to support the invertebrate mitochondrial genetic code. Subsequently, sequences of each sample were aligned against the reference IoA-00 mitochondrial genome using bowtie2 v2.2.6 [73]. Sequence variations were identified using GATK v3.5 [76] *HaplotypeCaller* function. SNPs common to all deltamethrin resistant individuals and lacking in all deltamethrin susceptible parasites were then identified.

### Phylogenetic reconstruction

The phylogenetic tree was constructed using full mtDNA sequences from sea lice strains IoA-00, IoA-01, IoA-02, IoA-03, NA01-P, NA01-O. Furthermore, hybrid F2 lice resulting from IoA-00 and IoA-02 crosses were included (for full pedigree, please see S5 Table), as well as sea lice caught from wild hosts. All sequence variations identified by the *HaplotypeCaller* function were collated with R/Bioconductor Package SNPRelate v1.6.3 [77]. An identity-By-State (IBS) analysis was conducted on the SNP genotypes to create a matrix of genome-wide average pairwise identities using the *snpgdsIBS* and *snpgdsHCluster* functions and a final phylogeny was reconstructed using the *snpgdsCutTree* function.

### Data access

The reference IoA-00 mitochondrial genome from this study have been submitted to the EBI Sequence Read Archive (SRA) study [project PRJEB15628]. The raw sequence traces and docker code used in this study are available at https://github.com/pseudogene/lice-mitochondria.

### Statistics

Statistical analyses were performed using the program Minitab 17.1.0 (Minitab Inc.). Median effective concentrations (EC_50_) of deltamethrin and cypermethrin effects on parasite behaviour and 95% confidence limits were calculated by probit analysis assuming a log-normal distribution of drug susceptibility. Gender and strain differences in drug susceptibility were assessed by comparing probit models of dose-response relationships. Total ATP levels were compared between control and drug exposed groups of *L*. *salmonis* using one way ANOVA (analysis of variance) followed by Dunnett’s test. Levene’s test was used to ascertain the homogeneity of variance in the data. The significance level was set at p < 0.05 in all tests.

## Supporting information

S1 Fig Actual deltamethrin concentration in *L*. *salmonis* bioassays. Bars show the average and standard deviation of results obtained for water samples taken at the beginning and the end of exposures (n = 3).
(PDF)

S2 Fig Geographic origin of salmon lice used to establish strains. Modified from a map created by N. Sinegina, used under a Creative Commons Attribution-Share Alike 4.0 License (http://www.supercoloring.com/silhouettes/scotland-map, accessed 9/5/2017).
(PDF)

S3 Fig Deltamethrin susceptibility of *L*. *salmonis* F1 parasites derived from crosses. Symbols show the average percentage of parasites (n = 10–15) that were rated “affected” after 30 min of exposure to indicated deltamethrin concentrations and 24 h of recovery.
(PDF)

S1 Table Bioassay raw data. The table compiles the raw data used to calculate EC_50_ values shown in Table 1.
(CSV)

S2 Table PCR primers to amplify *L*. *salmonis* mtDNA sequences. (CSV)

S3 Table Sequencing primers to sequence the above PCR products. (CSV)

S4 Table List of mtDNA sequencing reads. The table contains sample information and trimming coordinates for sequencing reads available under https://github.com/pseudogene/lice-mitochondria.
(CSV)

S5 Table Full pedigree of *L*. *salmonis* subjected to mtDNA sequencing. For parasites derived from the crosses show in Fig 1 that were subjected to mtDNA sequencing, information is provided about strain origin of P0 animals and pedigree of F1 and F2 animals.
(CSV)
