# Supplementary figures and images for: Maternal inheritance of deltamethrin resistance in the salmon louse Lepeophtheirus salmonis (Krøyer) is associated with unique mtDNA haplotypes
Source: PLoS One. 2017 Jul 12;12(7):e0180625. doi: 10.1371/journal.pone.0180625 (PMC5507548; doi:10.1371/journal.pone.0180625)

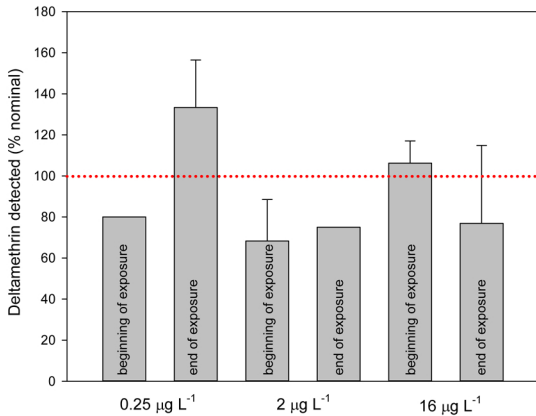

Supplement: S1 Fig — Bars show the average and standard deviation of results obtained for water samples taken at the beginning and the end of exposures (n = 3). (PDF) [file pone.0180625.s001.pdf]

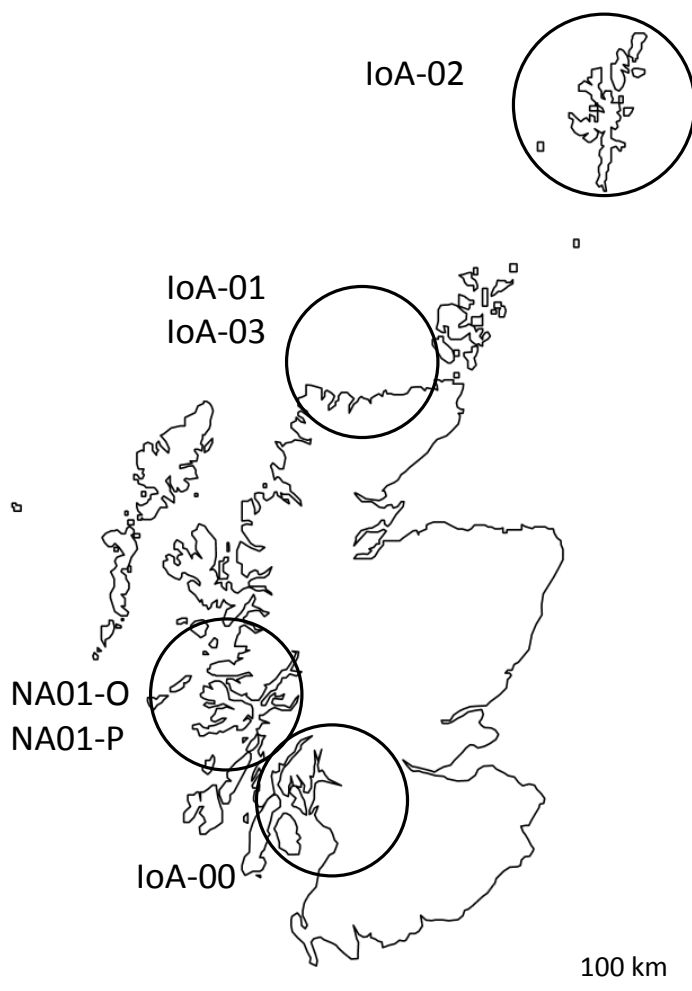

Supplement: S2 Fig — Modified from a map created by N. Sinegina, used under a Creative Commons Attribution-Share Alike 4.0 License (http://www.supercoloring.com/silhouettes/scotland-map, accessed 9/5/2017). (PDF) [file pone.0180625.s002.pdf]

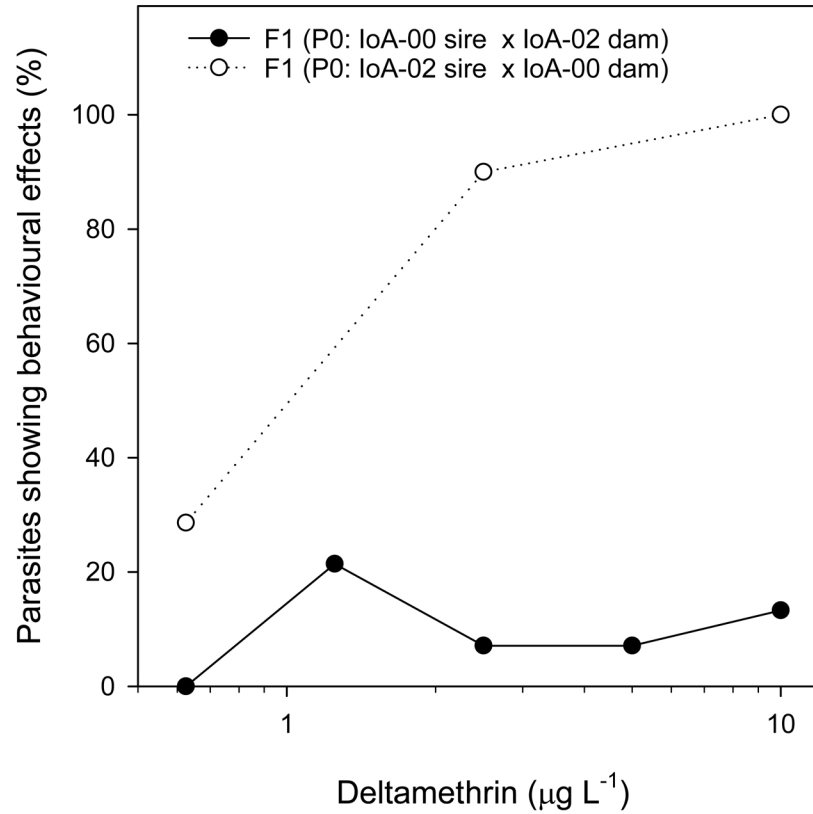

Supplement: S3 Fig — Symbols show the average percentage of parasites (n = 10–15) that were rated “affected” after 30 min of exposure to indicated deltamethrin concentrations and 24 h of recovery. (PDF) [file pone.0180625.s003.pdf]
